# Characteristics of Bacteriophage Isolates and Expression of Shiga Toxin Genes Transferred to Non Shiga Toxin-Producing *E. coli* by Transduction

**DOI:** 10.4014/jmb.2102.02040

**Published:** 2021-03-26

**Authors:** Da-Som Park, Jong-Hyun Park

**Affiliations:** Department of Food Science and Biotechnology, College of BioNano Technology, Gachon University, Seongnam 13120, Republic of Korea

**Keywords:** Bacteriophage, Shiga toxin, transduction, non-pathogenic *E. coli*, convertant

## Abstract

A risk analysis of Shiga toxin (Stx)-encoding bacteriophage was carried out by confirming the transduction phage to non-Stx-producing *Escherichia coli* (STEC) and subsequent expression of the Shiga toxin genes. The virulence factor *stx1* was identified in five phages, and both *stx1* and *stx2* were found in four phages from a total of 19 phage isolates with seven non-O157 STEC strains. The four phages, designated as φNOEC41, φNOEC46, φNOEC47, and φNOEC49, belonged morphologically to the Myoviridae family. The stabilities of these phages to temperature, pH, ethanol, and NaClO were high with some variabilities among the phages. The infection of five non-STEC strains by nine Stx-encoding phages occurred at a rate of approximately 40%. Non-STEC strains were transduced by Stx-encoding phage to become lysogenic strains, and seven convertant strains had *stx1* and/or *stx2* genes. Only the *stx1* gene was transferred to the receptor strains without any deletion. Gene expression of a convertant having both *stx1* and *stx2* genes was confirmed to be up to 32 times higher for Stx1 in 6% NaCl osmotic media and twice for Stx2 in 4% NaCl media, compared with expression in low-salt environments. Therefore, a new risk might arise from the transfer of pathogenic genes from Stx-encoding phages to otherwise harmless hosts. Without adequate sterilization of food exposed to various environments, there is a possibility that the toxicity of the phages might increase.

## Introduction

Shiga toxins (Stx) are a group of bacterial toxins those cause human and animal diseases. Stx is produced primarily by *Escherichia coli* and *Shigella dysenteriae*, and sporadically by *Citrobacter freundii*, *Enterobacter cloacae*, and *S. flexneri* [1]. Stx has two major types: Shiga toxin1 (Stx1) and Shiga toxin2 (Stx2). Stx1 and Stx2 have very similar biochemical properties and mechanisms, however, their immunological characteristics are different. Clinically, Stx2 is approximately 1000 times more toxic than Stx1 [2, 3].

There are approximately 200 serotypes of Stx-producing *E. coli* (STEC). Typical examples are *E. coli* O157:H7 and non-O157 STEC serotypes, including O26, O103, O111, O118, O145, and O156. Strains O103 and O111 account for approximately 50% of total non-O157 STECs [4]. STEC pathogenesis is caused by various virulence factors encoded in the genome, plasmids, and bacteriophages [5, 6]. STEC causes hemorrhagic colitis (HC) by adhering to the intestinal mucosa and producing Stx. Severe diseases such as hemolytic uremic syndrome (HUS) can be induced in children and the elderly, and have been reported worldwide [7, 8].

Bacteriophages (phages) containing the *stx* gene play a major role in mediating pathogenicity, by transferring pathogenetic genes to generic *E. coli*. Phages have been found in soil, sewage, rivers, and feces. Pathogenic genes may be acquired from other phages [1, 9]. Stx is encoded in the late gene region of the lysogenic phage, which is expressed only when the phage in the host cell is induced by replication or the lytic cycle [10, 11]. STEC can be exposed to various environmental stresses, such as heat, antibiotics, and UV. Phages are induced and pathogenic gene transfer to other bacteria may be possible in such environments [12, 13]. It has been reported that hydrogen peroxide treatment also induces the induction of Stx-encoding phage. This observation suggests that the hydrogen peroxide in fermented foods may induce Stx-encoding phages and promote gene transfer [14].

It has recently been confirmed that Stx-encoding phages are also induced by high hydrostatic pressure, which is widely used for food preservation. Phages may be induced, and the transfer of pathogenic genes to non-pathogenic *E. coli* may be facilitated, if treatments designed to ensure the microbiological safety of foods are not performed correctly [15]. Stx-encoding phages can also be induced spontaneously without external induction, indicating that free Stx-encoding phage may appear easily [16, 17].

In this study, Stx-encoding phage was isolated from various environmental samples and transfer of the Shiga toxin gene to non-STEC was confirmed through transduction and expression to investigate the risk of phages in the food chain.

## Materials and Methods

### Isolation and Purification of the Phages

To isolate phages, seven non-O157 Stx *E. coli* (STEC) strains from the National Culture Collection for Pathogens (NCCP) were used as host strains: *E. coli* NCCP13937 (serotype O103), *E. coli* NCCP14018 (O91), *E. coli* NCCP13970 (O111), *E. coli* NCCP13934 (O179), *E. coli* NCCP 13979 (O104), *E. coli* NCCP13987 (O55), and *E. coli* NCCP14010 (O26).

Twelve samples of soil, river water, and domestic sewage were collected from the Seongnam Water Reclamation Center (Korea). The samples were diluted 10 times with LBC broth (Luria Bertani broth + 10 mM CaCl_2_, Difco Laboratory, USA) and homogenized. The host cultures were inoculated and cultured to a level of 8-9 log CFU/ml at 37°C at 150 rpm (Jeiotech, Korea) for 24 h. After centrifugation at 10,000 ×*g* for 10 min, the culture supernatants were filtered through a 0.22 μm syringe filter (Millipore, USA). The sterilized supernatants were mixed with 0.6%LBC soft agar and the host strains. Plaque assays were performed using the double overlay agar method on LBC agar and cultured at 37°C for 24 h [18]. The phages from the plaques were separated, purified, and stored in 10%glycerol stock at -80°C in a deep freezer. Purification of the phages was performed using CsCl gradient centrifugation [19]. Concentrated phage was applied to the step gradient solution by CsCl densities of 1.3, 1.45, 1.5, and 1.7 g/cm^3^ and centrifugated by 78,500 ×*g* for 2 h (Optima L-100XP, USA). The fraction of 1.45 g/cm^3^ density was taken by a syringe and dialysed with SM buffer [100 mM NaCl, 10 mM MgSO_4_, 50 mM Tris-HCl (pH 7.5), 0.01% gelatin?

### Differentiation and Identification of the Phage Isolates

For differentiation of the isolated phages, tricine SDS-PAGE gels were used to analyze the structural protein patterns. Purified phage solution (16 μl, approximately 9-10 log PFU/ml) and 4 μl of 5× sample buffer (T&I, Korea) were mixed and boiled for 10 min. Gel electrophoresis was performed at 120 V for 60 min. Staining was performed for 1 h with a staining reagent (0.2% w/v Coomassie blue (R-250, T&I), 10% v/v acetic acid, 50% v/v CH_3_OH, 40% v/v D.W) and decolorized with the reagent (40% v/v methanol, 10% v/v acetic acid, 50% v/v D.W)[20].

Phage DNA was extracted to analyze restriction enzyme patterns. The phage was concentrated to 9-10 log PFU/ml with 20% polyethyleneglycol 80 and treated with DNase and RNase (Sigma-Aldrich, USA). Again after treating with proteinase K (Sigma-Aldrich), the lysis buffer [0.5 M EDTA, 10% w/v SDS, 1 M Tris (pH 8.0)] was applied. The proteins and impurities were removed with phenol : chloroform : isoamylalcohol (Sigma-Aldrich), washed with ethanol, and dissolved in diethylpyrocarbonate water (Bioneer, Korea) for use. The extracted DNA and restriction enzymes *SalI* and *SacII* (Takara, Japan) were reacted at the optimal temperature and time for each enzyme. The cleaved phage DNA was confirmed by electrophoresis on a 1% agarose gel [21].

Transmission electron microscopy (TEM) was used to analyze the morphological characteristics of Stx-encoding phages. The purified solution was attached to a carbon-coated copper grid (200 mesh, Ted Pella, USA) for 2 min, washed with sterile distilled water, and dehydrated. It was then stained with an equal volume of 2%uranyl acetate for 30 s, washed with distilled water, and dried at room temperature. Negative staining was performed and observed at a magnification of 30,000 at a voltage of 80 kV using a TEM (H-7600, Hitachi, Japan).

### Virulence factor Identification and One-Step Growth of the Phages

To analyze the virulence factor profile of the phages, specific primers for *stx1*, *stx2*, enterohemolysin-encoding gene (*ehxA*), autoagglutinating adhesion-encoding gene (*saa*), and attaching/effacing protein-encoding gene (*eae*) were used, and the results were confirmed by electrophoresis. The primers and PCR conditions were as previously described [22-26].

The host was cultured to 8-9 log CFU/ml in LBC broth, mixed at a multiplicity of infection (MOI) of 10^-5 with the phages, and incubated at 37 °C for 10 min with shaking at 150 rpm. Centrifugation at 10,000 ×*g* for 10 min was performed to remove free phage particles from the supernatant. Pellets were suspended in 10 ml of fresh LBC broth, and 100 μl aliquots were taken for the plaque assay. After incubation for 24 h at 37 °C, plaque counts were measured. The latent periods and burst sizes were determined according to Hyman *et al*. [26].

### Stability Analysis of the Phages

To investigate the stability of the phages at high temperature, 100 μl of concentrated phage solution (~ 11 log PFU/ml) was added to a 1.5-ml microtube and incubated at 65-75°C for 30 min using a heat block. A plaque assay for the heated phage was performed using the double overlay method. The number of plaques was counted and compared to that in the unexposed control group. For pH stability, the phage solution (10 μl) was mixed with 990 μl of SM buffer (100 mM NaCl, 10 mM MgSO_4_, 50 mM Tris-HCl, pH 7.5) adjusted to pH 2-10 with HCl and NaOH. After incubation at room temperature for 30 min or 1 h, plaque assays were performed using the double overlay method. To investigate the stability of the phages in organic solvents, the final concentration of each alcohol was adjusted to 30-70% by mixing absolute ethanol (Georgia Chem, USA) with the phage, and incubating at room temperature for 30 min and 1 h. Plaque assays were performed using the double overlay method. To investigate the stability of the phages under sodium hypochlorite, the final concentration of sodium hypochlorite was adjusted to 100-500 ppm by mixing 6-14% sodium hypochlorite (Korea) with the phage solution and incubating at room temperature for 30 min or 1 h. Plaque assays were performed using the double overlay method.

### Phage Transduction to non Stx-Producing *E. coli* (STEC) and Lysogenic Cell Preparation

Spot assays were performed to analyze the host infection of nine Stx-encoding phages to the five non-STEC strains of *E. coli* ATCC 9637, *E. coli* ATCC 8739, *E. coli* ATCC 10536, *E. coli* ATCC 11775, and *E. coli* ATCC 25922. Plaques were confirmed using spot assays, and the range of host infection was determined.

A phage lysate solution was prepared to produce lysogenic bacteria. One milliliter of LBC broth was inoculated with 10 μl of five non-STEC cultures (8-9 log CFU/ml) and incubated at 37°C for 30 min. Ten microliters of phage (8-9 log PFU/ml) was added to the host solution and incubated at 37°C for 2 h. After incubation, 0.1 ml chloroform was added, the solution was vortexed vigorously, centrifuged at 14,000 ×*g* for 1 min, and the supernatant was transferred to a sterile tube. After treatment with chloroform, the solution was used as a phage lysate solution. The method of Bielaszewska *et al*. [4] was modified and used for further processing. One milliliter of non-STEC culture was centrifuged at 14,000 ×*g* for 1 min. The pellet was suspended in 500 μl of CaMg (5 mM CaCl_2_, 10mM MgSO_4_) to make a host solution [4]. The solution and phage lysate solution (1:1) were mixed and incubated at room temperature for 30 min. LB broth was added and incubated at 37°C and 150 rpm for 1 h. After centrifugation at 14,000 ×*g* for 1 min, the pellet was suspended in 1 ml of LBC broth, diluted, and spread on an LBC plate. The plate was incubated at 37°C for 24 h. A colony from each plate was randomly selected and subcultured three times on LBC agar to identify stable lysogenic bacteria. After DNA extraction, PCR with *stx1* and *stx2* specific primers was performed to confirm whether the convertant cells had the genes.

### Expression of stx Genes on the Convertant Strain

To confirm the expression of *stx* genes in the convertant strains, 100 μl of convertant solution was inoculated in 5 ml CA-YE broth (Himedia,) supplemented with NaCl from 1% to 10% and incubated at 37°C. The supernatant was centrifuged at 1,500 ×*g* at 4°C for 20 min and refrigerated for verocytotoxin analysis. VTEC-reverse passive latex agglutination toxin detection kits (Denka Sieken Co., Ltd., Japan) were used according to the proposed method [27]. In a 96-well V-bottom microtiter plate, the supernatant was mixed with the supplied diluent at 1 : 1 (25 μl), and the same volume of latex particles sensitized with rabbit polyclonal anti-VT1 or anti-VT2 immunoglobulin G antibody was added. The plates were covered and reacted at room temperature for 20 h, and latex agglutination was examined. The toxins provided with the kit for positive and negative controls were used for each analysis.

## Results and Discussion

### Phage Isolation and Virulence Factor Identification

A total of 19 phages from 12 samples were isolated from seven non-O157 STEC host strains (Table 1). The phages were selected from different plaques, even in the same samples, and then isolated to purify them. Fifteen phages (79%) were isolated from seven sewage samples, and four phages (21%) were isolated from river water samples. The phages were named ϕNOEC, followed by numbers. Municipal wastewaters and activated sludge contained 8-9 log virus particles/ml, which is the highest concentration among the environments, and the next highest was by marine environments [28-30]. The ratio of virus to bacterial cells in wastewater is approximately 10:1. A total of 55 phages were isolated from the environmental samples using two *E. coli* O157:H7 and six non-O157 *E. coli* as host strains [31]. These results indicate that there are many phages in the environment.

To identify the virulence factors of the phages, PCR was performed using *stx1*, *stx2*, *ehxA*, *saa*, and *eae*-specific primers. For *stx1* and *stx2*, three phages (15%) had only *stx1*, and two phages (10%) had only *stx2*. Four phages (21%)—ϕNOEC41, ϕNOEC46, ϕNOEC47, and ϕNOEC49—had both *stx1* and *stx2*, which were focused on further studies. The other virulence factors, *ehxA*, *saa*, and *eae*, were detected in the same proportion as stx. Stx2-encoding phages are widely distributed, and were found to be 32-94% in the environmental samples polluted with feces; however, only 1% of the phages isolated from wastewater or river water had both *stx1* and *stx2* [32, 33]. Therefore, the Shiga-toxin virulence factor of the phages was distributed widely and in large numbers. As well as *stx1* and *stx2*, the phages also had other virulence factors, and could be considered to be highly harmful.

### Structural Characterization of Stx-Encoding Phages

**Fig. 1:**
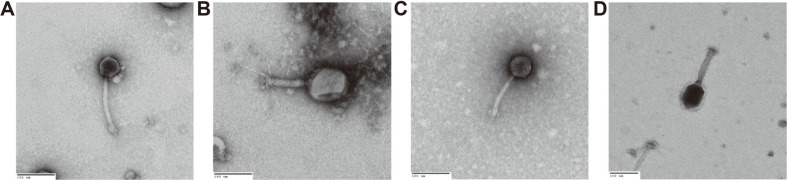
Morphological characteristics of Stx-encoding phages by TEM for ϕNOEC41 (A), ϕNOEC46 (B), ϕNOEC47 (C), and ϕNOEC49 (D). Size bar,100 nm; Magnification, ×30,000.

The structural difference of ϕNOEC41, ϕNOEC46, ϕNOEC47, and ϕNOEC49 were investigated using the restriction enzymes *SalI*, *EcoRI*, and *SacII*. SDS-PAGE confirmed that they had different genome structures and protein compositions (Fig. S1). The morphological characteristics of the Stx-encoding phages were analyzed using transmission electron microscopy (TEM). The Stx-encoding phages ϕNOEC41, ϕNOEC46, ϕNOEC47, and ϕNOEC49 belonged to the Myoviridae family (Fig. 1). ϕNOEC41 had a head diameter of 48 nm, the smallest in the group, and its tail length was 150 nm. The head diameter of ϕNOEC46 was 94 nm, the largest in the group, and its tail length was 121 nm. ϕNOEC47 and ϕNOEC49 were similar, and had dimensions intermediate between those described above.

One-step growth analysis was performed for four Stx-encoding phages. The latent period was 15-25 min, one cycle duration was 25-30 min, and the rise period was 10-15 min, The burst size was approximately 33-441 PFU/infected cells of various sizes. The non-O157 STEC phage group was reported to have a latent period of 25-40 min, a cycle of 45-70 min, and a burst size of 40-176 PFU/infected cells [31]. The isolated non-O157 STEC phage had a burst period of 8-37 min, a burst size of 12-794 PFU/infected cells, and a 19-40 min rise period [34]. Therefore, the characteristics of Stx-encoding phages identified using the one-step growth curve varied, but were similar to those of the previous reported phages.

### Stability of Stx-Encoding Phages

ϕNOEC41 and ϕNOEC47 were more stable, with a reduction of 3.6 log PFU/ml and 1.0 log PFU/ml after exposure to 70oC for 30 min, respectively than ϕNOEC46 and ϕNOEC49. ϕNOEC47 showed the highest stability, decreasing by only 2.5 log PFU/ml even at 75°C (Fig. S2-A).

Four Stx-encoding phages showed low stability at pH 2. ϕNOEC41 and ϕNOEC47 showed a maximal decrease of 5.2 log PFU/ml and 6.4 log PFU/ml, respectively (Fig. S2-B). At pH 3-10, the four phages showed a decrease of less than 3 log PFU/ml and were relatively stable, and were similar in same temperature and acidic conditions [31, 34]. Therefore, the stabilities in high temperature and acidic environments were somewhat different among the phages, but all were similar to those previously reported [31, 34].

The phages showed a gradual decrease in count in response to increasing ethanol concentration after treatment for 30 min (Fig. S3). ϕNOEC49 showed a large decrease of up to 4 log PFU/ml at 50%-70% ethanol, and was found to be relatively unstable in ethanol. The Stx2-encoding phage decreased by approximately 6 log PFU/ml in 70%ethanol, and was reported to be unstable [35]. Three non-O157 STEC phages were exposed for 1 h under the same conditions and decreased to 3 log PFU/ml at 30%, 50%, and 70% [31]. Therefore, two of the three Stx-encoding phages were relatively stable in ethanol.

The phages ϕNOEC41, ϕNOEC46, ϕNOEC47, and ϕNOEC49 showed a decrease of only one PFU/ml, indicating very high stability at various concentrations of NaClO (Fig. S3). There was a slight difference in stability among the phages. The stability in sodium hypochlorite was high even at high concentrations of sodium hypochlorite. The STEC phage showed a reduction of 1.1 log PFU/ml when treated with 20 ppm chlorine for 30 min [36]. Stx-encoding phages have been shown to be very stable under food-processing conditions [13, 15, 33, 35, 37]. Therefore, phages with high resistance to inactivation factors might be appropriate candidates for the transfer of toxin genes.

### Transduction of Shiga Toxin Phage to Non-STEC and Lysogenic Convertants

To analyze the host infection of Stx-encoding phages, the plaques were identified by spot assay on five non-STEC type strains (Table 2). ϕNOEC43 and ϕNOEC49 infected four of the five strains at a rate of 80%. However, the three phages ϕNOEC41, ϕNOEC45, and ϕNOEC47 showed no infection on any strain. Overall, host infectivity by phage was observed in 18 of 45 (40%) hosts.

Lysogenic cells for five non-STEC hots were prepared with above phage lysates. Infection occurred in seven of the 18 (39%) hosts sabilized. PCR was performed using a stx-specific primer (Table 3). Transduction of ϕNOEC36, ϕNOEC40, and ϕNOEC43, which had only *the stx1* gene, was confirmed without loss of the gene in *E. coli* ATCC 9637 and *E. coli* ATCC 11775. Transduction of ϕNOEC46 and ϕNOEC49, which had the *stx1* and *stx2* genes, led to the gain of *stx1* and sometimes loss of the *stx2* gene. There was one *E. coli* ATCC 25922 convertant cell with both *stx1* and *stx2*, which was lysogenized by ϕOEC49. Thus, transfer of the *stx2* gene is limited during transduction in some strains. Transduction of pathogenic gene-carrying phages is suggested to occur in 29% and 30% of the receptor strains for non-pathogenic *E. coli*, and to be a lysogenic bacterium, capable of transferring toxic genes [4, 38]. Thus, the presence of temperate phage in the environment would present a hazard for food-borne poisoning by means of transduction in the food chain.

### Expression of Toxic Genes of the Convertant *E. coli* under Saline Conditions

**Fig. 2:**
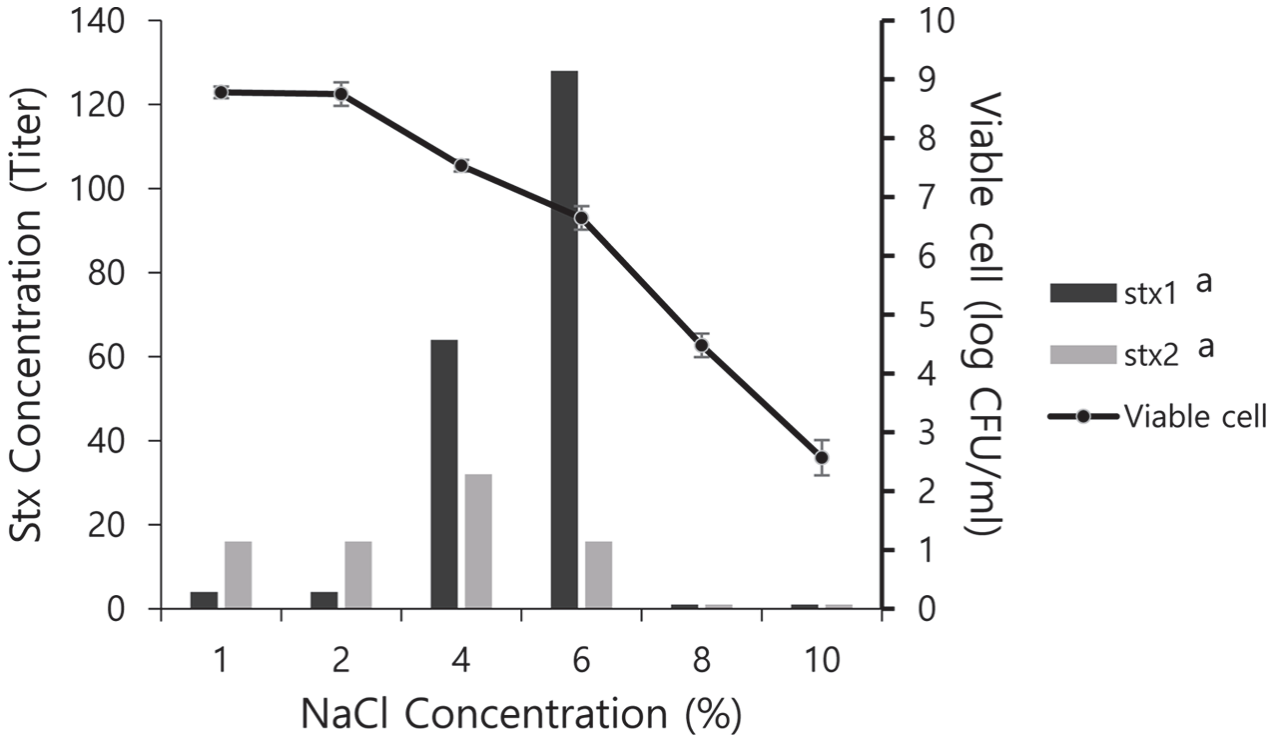
Growth and Shiga toxin production of *E. coli* ATCC 25922 (ϕNOEC49). Growth from 2.5 to 8 CFU/ml was done under various NaCl concentrations and the experiment was repeated by three times.

The conversion of bacteria by the transduction of the phages might contaminate foods and be exposed to the osmotic pressures of various food components. Convertant *E. coli* ATCC 25922 (ϕNOEC49) was cultured under osmotic stress conditions on LBC media with 1-10% salt to confirm the expression of stx toxin genes. The convertant grew well from 2.5 log CFU/ml to 6-8 log CFU/ml at a concentration of 6% NaCl (Fig. 2). Using the VTEC-RPLA toxin detection kit, four titers of Stx1 and 16 titers of Stx2 toxins were expressed in the culture solution of 8 log CFU/ml in media with 1-2% salt. At 4% salt, 64 titers of Stx1 and 32 titer of Stx2 toxins on 7 log CFU/ml were expressed. For a higher salt concentration (6%), the highest concentration of Stx1 was expressed by 128 titers, and 16 titers of Stx2 toxin were expressed at 6 log CFU/ml. Stx1 and Stx2 toxins were not expressed at 2-4 log CFU/ml under conditions of 8% and 10% salt. The *stx1* gene was highly expressed in proportion to the salt concentration, and was 32 times higher in 6% salt than that in low-salt conditions. Shiga toxin producing *E. coli* grows in 5.25% salt media, so the characteristics of the convertant may be related to cell growth [39]. The transcription of *stx1*A and *stx2*A in enterohemorrhagic *E. coli* was six and two times higher, respectively, than under low-salt conditions, indicating that high transcription of the toxin genes is induced in response to long-term osmotic stress produced by a salt concentration of 4.5% [40].

In conclusion, Stx-encoding phages isolated from the environment were found to be highly stable under extreme conditions. The Shiga toxin genes of phage could be transferred to non-toxigenic *E. coli* by transduction, and could be expressed in the convertant cell. Therefore, the transfer of pathogenic genes from Stx-encoding phages may pose a risk to human health. In the case of inadequate sterilization of food exposed to various environments, there is a possibility that the toxicity of the temperate phages might increase.

## Supplemental Materials



Supplementary data for this paper are available on-line only at http://jmb.or.kr.

## Figures and Tables

**Table 1:** Phage isolates and distribution of virulence genes in the environmental samples.

Hosts	Phages	Virulence factors				

		*stx1*	*stx2*	*ehxA*	*saa*	*eae*
*E. coli* NCCP 13934^*^	ϕNOEC31	-	-	-	-	-
*E. coli* NCCP 13937	ϕNOEC32	-	-	-	-	-
*E. coli* NCCP 13987	ϕNOEC33	-	-	-	-	-
*E. coli* NCCP 13970	ϕNOEC34	-	-	-	-	-
*E. coli* NCCP 14010	ϕNOEC35	-	-	-	-	-
*E. coli* NCCP 14018	ϕNOEC36	+	-	+	+	+
*E. coli* NCCP 13934	ϕNOEC37	-	+	+	+	+
*E. coli* NCCP 14010	ϕNOEC38	-	-	-	-	-
*E. coli* NCCP 14018	ϕNOEC39	-	-	-	-	-
*E. coli* NCCP 13937	ϕNOEC40	+	-	+	-	+
*E. coli* NCCP 13979	ϕNOEC41	+	+	-	+	-
*E. coli* NCCP 13979	ϕNOEC42	-	-	-	-	-
*E. coli* NCCP 13934	ϕNOEC43	+	-	-	-	-
*E. coli* NCCP 13937	ϕNOEC44	-	-	-	-	-
*E. coli* NCCP 13979	ϕNOEC45	-	+	-	-	-
*E. coli* NCCP 13934	ϕNOEC46	+	+	+	+	+
*E. coli* NCCP 13979	ϕNOEC47	+	+	-	+	+
*E. coli* NCCP 13979	ϕNOEC48	-	-	-	-	-
*E. coli* NCCP 13934	ϕNOEC49	+	+	+	+	+
Total	19	7	6	5	6	6

^*^NCCP: National Culture Collection for Pathogens Symbols: +; detected, −; not detected

**Table 2:** Infection spectrum of Stx-encoding phages to non Shiga toxin-producing *E. coli*.

Stx-encoding phages	Non-STEC host				

	*E. coli* ATCC 9637	*E. coli* ATCC 8739	*E. coli* ATCC 10536	*E. coli* ATCC 11775	*E. coli* ATCC 25922
ϕNOEC36	+	+	+	-	-
ϕNOEC37	+	-	+	-	-
ϕNOEC40	+	-	+	-	-
ϕNOEC41	-	-	-	-	-
ϕNOEC43	+	+	+	+	-
ϕNOEC45	-	-	-	-	-
ϕNOEC46	+	-	+	+	-
ϕNOEC47	-	-	-	-	-
ϕNOEC49	+	+	+	-	+

Symbols: +, plaque detected; −, plaque not detected

**Table 3:** Convertant cells by phage stx-gene transfer to non Shiga toxin *E. coli* host strains.

Host and convertant *E. coli*	*stx1*	*stx2*
*E. coli* ATCC 9637	-	-
*E. coli* ATCC 9637 (ϕNOEC36)	+	-
*E. coli* ATCC 9637 (ϕNOEC40)	+	-
*E. coli* ATCC 9637 (ϕNOEC46)	+	-
*E. coli* ATCC 9637 (ϕNOEC49)	+	-
*E. coli* ATCC 11775	-	-
*E. coli* ATCC 11775 (ϕNOEC43)	+	-
*E. coli* ATCC 11775 (ϕNOEC46)	+	-
*E. coli* ATCC 25922	-	-
*E. coli* ATCC 25922 (ϕNOEC49)	+	+

Symbols: +, detected; −, not detected
