# Supplementary material for: Characteristics of Bacteriophage Isolates and Expression of Shiga Toxin Genes Transferred to Non Shiga Toxin-Producing E. coli by Transduction
Source: J Microbiol Biotechnol. 2021 Mar 26;31(5):710–6. doi: 10.4014/jmb.2102.02040 (PMC9705938; doi:10.4014/jmb.2102.02040)
Supplement: Supplementary file 1 [file jmb-31-5-710-supple.pdf]

J. Microbiol. Biotechnol.
https://doi.org/10.4014/jmb.2102.02040

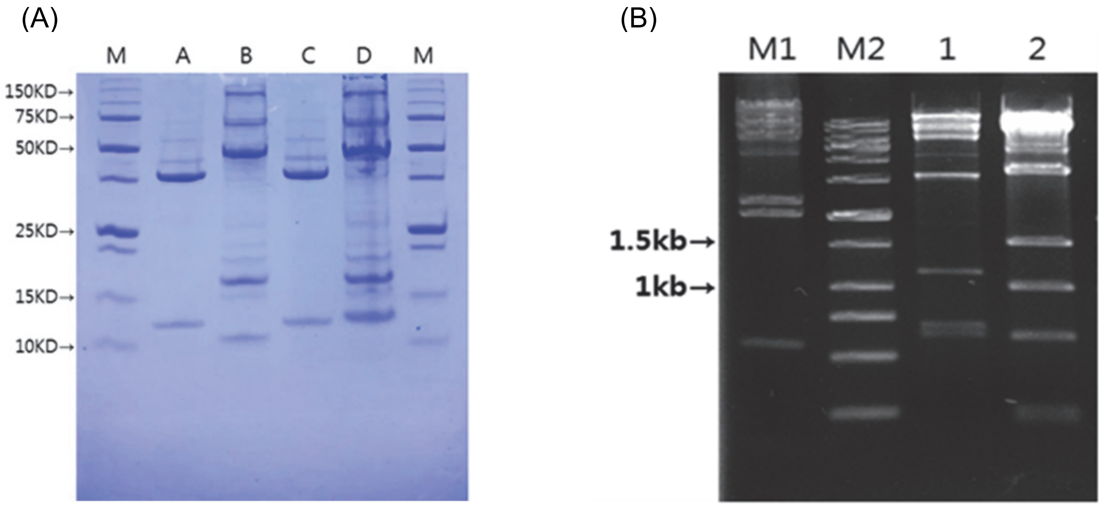

Supplementary Fig. 1. Analysis of Stx-encoding phage difference by SDS-PAGE (A) and restriction enzyme of *Sal* I (B). Symbols: (A) M, protein marker; A, ϕNOEC41; B, ϕNOEC46; C, ϕNOEC47; D, ϕNOEC49, (B) M1, Lambda DNA ladder; M2, 1kb DNA ladder; 1, ϕNOEC41; 2, ϕNOEC47

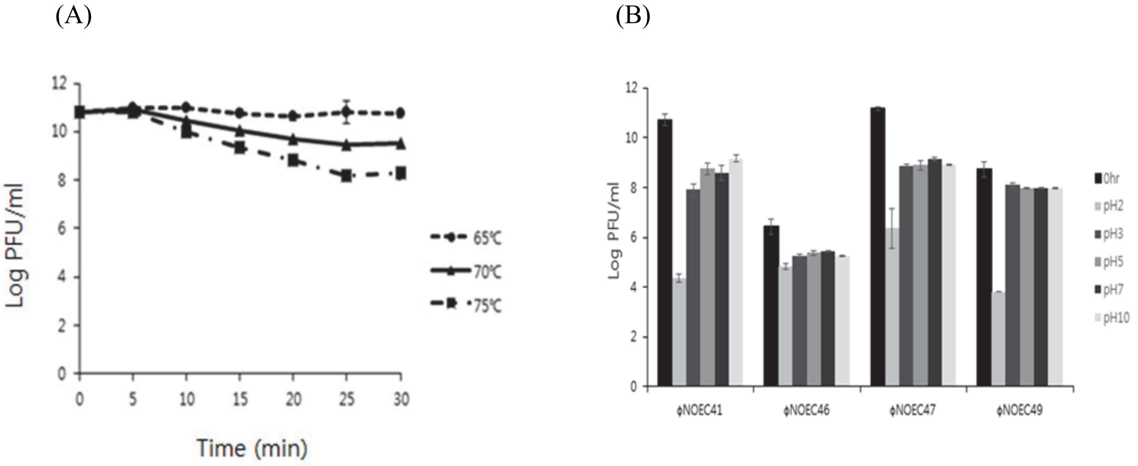

Supplementary Fig. 2. Stabilities of the pages under the various temperature and pH for ϕNOEC47 (A) and Stx-encoding phages (B) in 1 h.

(A)

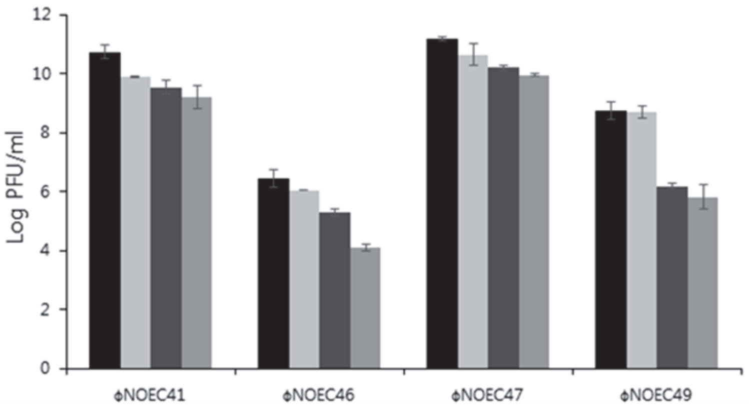

(B)

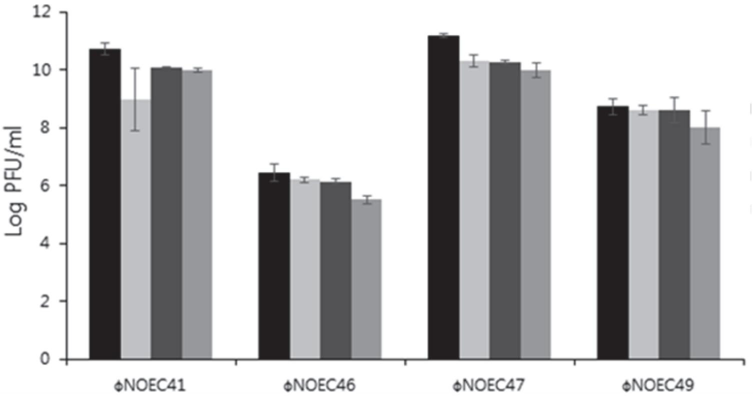

Supplementary Fig. 3. Stabilities of Stx-encoding phages under various concentrations of EtOH (A) and NaClO (B) for 30 min at room temperature. Symbols: black rectangular (0 h); light grey rectangular (A-30%, B-100 ppm); dim grey rectangular (A-50%, B-200 ppm); grey rectangular (A-70%, B-500 ppm) in sequence.
